# Partisan differences in healthcare decision-making: Evidence from a vaccine experiment

**DOI:** 10.1371/journal.pone.0352319

**Published:** 2026-07-20

**Authors:** Brandyn F. Churchill, Xiaoxue Sherry Gao, Rong Rong

**Affiliations:** 1 Department of Public Administration & Policy, American University, District of Columbia, United States of America; 2 Department of Resource Economics, University of Massachusetts Amherst, Amherst, Massachusetts, United States of America; Leiden University: Universiteit Leiden, NETHERLANDS, KINGDOM OF THE

## Abstract

Policymakers use information campaigns to alter health behaviors, but their effectiveness depends on how the information compares to viewers’ initial beliefs and their perceptions of its value. In August 2024, we experimentally tested whether political identity influenced how individuals responded to information about the COVID-19 vaccine. Our sample included 288 self-identified Democrats and 291 self-identified Republicans. We found that Democrats and Republicans similarly overestimated the frequency and severity of COVID-19 infections, as well as the vaccination rate. Participants were not better at predicting the COVID-19 experiences of their concordant party. However, participants who learned they underestimated the discordant party vaccination rate were more likely to report an intent to receive a COVID-19 vaccine.

## Introduction

In the United States, Democrats and Republicans strongly disagreed on the severity of the COVID-19 pandemic [1–4] and the appropriateness of government interventions intended to mitigate it [5,6]. A particularly contentious areas of partisan disagreement involved government efforts to increase take-up of the COVID-19 vaccine [7,8], including through vaccine subsidies [9], mandates [10], and promotional campaigns [11,12]. Today, partisan affiliation is a stronger predictor of COVID-19 vaccination than education, race/ethnicity, or health insurance coverage [13,14].

This paper explores the importance of political identity in vaccination within the United States. First, we conducted a survey to learn about the COVID-19 experiences of Democrats and Republicans. Second, we used an online research platform to elicit health beliefs from 579 participants (288 Democrats and 291 Republicans). This sample was designed to be politically representative by age and sex [15]. Approximately half the participants were asked about the experiences of Democrats, while the other half were asked about the experiences of Republicans. For each participant we randomly selected one of the elicited beliefs and revealed the share from the pre-experiment survey. We conceptualize this reveal as an “information shock” depending on (i) the participants’ initial beliefs and (ii) the concordance between their political affiliations and the political identity serving as the basis for the reveal [16]. In this sense, we allow the relative importance of the difference between the revealed value and the initial health belief to vary by partisan concordance, given that people have differing expectations about the health behaviors of members of their own political party and members of the opposing political party [17–21].

Despite widespread use, it is not theoretically clear whether information campaigns will achieve their intended effects. Existing work suggests that information campaigns often have modest effects [22,23]. For one, the size of the effect depends on the medium through which the message was conveyed [24] and the degree to which recipients trust the source of the information [25,26]. For example, several studies have shown that Republicans are more inclined to trust vaccine messaging from high-profile Republican leaders, such as President Trump [27–29]. Additionally, it is possible for an information campaign to have an effect opposite its intended if it corrects the initial beliefs of individuals who had previously overestimated the risk of the targeted behavior [30–32]. For example, consider two individuals deciding whether to receive a COVID-19 vaccine. The first person believes that 10% of people will contract the disease, while the second person believes that 40% of people will contract it. A promotional campaign stating that 20% of people contract COVID-19 serves as a positive information shock to the first person but a negative information shock to the second person, resulting in an unclear effect on vaccination.

A key contribution of our study is to document substantial variation in participants’ beliefs regarding COVID-19, resulting in potential differential effects of information campaigns. On average, we show that Democrats and Republicans similarly overestimated the frequency and severity of COVID-19 infections and underestimated the vaccine side effect rate. [33] also found individuals overestimated their COVID-19 risk. While participants held accurate beliefs regarding the share of Democrats vaccinated against COVID-19, members of both parties overestimated the share of vaccinated Republicans. Interestingly, there is little evidence that participants were better at predicting the experiences of members of their concordant party. While the experimental results are mostly inconclusive, we consistently find that participants who underestimated (overestimated) the share of discordant party vaccinated against COVID-19 were subsequently more (less) likely to report an intent to vaccinate. Placebo and falsification exercises indicate that this result is unlikely to have occurred by chance.

We build on work studying the effects of partisan differences on health [34–38]. We also add to a body of evidence exploring the economic determinants of vaccination [39–44]. While messaging emphasizing personal benefits is predictive of vaccination [45,46], the efficacy may depend on whether viewers relate to the messenger. [26] showed that racially concordant non-experts were most effective at promoting influenza and COVID-19 vaccination intent among Black men without a college degree. Researchers have examined the effects of President Trump’s messaging on vaccination [27–29,47,48], though this work does not separate celebrity endorsement [49–51] from political identity.

## Experimental design and empirical approach

### Pre-experiment survey

We asked our experimental participants about the COVID-19-related experiences of Democrats and Republicans. Because data on the COVID-19 experiences included in our study are not consistently available by partisan affiliation, we conducted a pre-experiment survey via an online research platform, Prolific. Prolific is increasingly being used by researchers (see, for example, [52–55]) because it produces higher quality data than other popular online research platforms, such as Amazon’s Mechanical Turk [56–58]. Because infections spike in autumn and winter months [59–61], we collected responses during 05/12/2024-05/16/2024 when individuals were likely to remember their recent experiences. The survey was estimated to take 10 minutes, and participants earned $3 (hourly wage of $18); the monetary incentive was provided to encourage higher effort from the respondents [62]. The survey is included as supporting information.

We asked respondents five questions about their COVID-19 experiences during the past 12 months, including (i) whether they had tested positive for COVID-19 (either using an at-home test or at a physician’s office), (ii) whether they had required medical intervention for a COVID-19 infection (either visiting a physician to address the symptoms or receiving an anti-viral prescription to treat the infection), (iii) whether they had received a COVID-19 vaccine (either an initial shot or a booster), (iv) whether they had experienced side effects from vaccination, and (v) to rank seven potential reasons they chose to receive a COVID-19 vaccine from 1 to 5, with larger numbers indicating higher importance and ties permitted. We provided individuals with a list of common side effects compiled by Centers for Disease Control and Prevention (https://www.cdc.gov/vaccine-safety/vaccines/covid-19.html) and allowed individuals to list additional side effects. Only those who reported an infection were asked whether they required medical intervention. Likewise, only respondents who reported vaccinating were asked whether they had experienced side effects and about their motivation. We classified individuals as vaccinating to protect others if they ranked “Protecting Others Within My Household” or “Protecting Others Outside My Household” among the most important reasons for vaccinating. The experiment was approved by the Institutional Review Board at the University of Massachusetts Amherst under protocol #5397. Consent was collected electronically. The full survey instrument is reproduced in S1 Text.

Table 1 reports the shares from our pre-experiment survey (Panel A). Democrats and Republicans reported similar infection and severity rates. Yet there are stark partisan differences in vaccination. While 46% of Democrats reported receiving a recent COVID-19 vaccine, only 28% of Republicans reported doing the same. Among those receiving a COVID-19 vaccine, over 80% of both Democrats and Republicans reported experiencing side effects. Finally, we find that 72% of vaccinated Democrats and 64% of vaccinated Republicans said they primarily did so to protect others.

**Table 1:** Experiences of 100 Democrats and 100 Republicans with COVID-19.

	(1)	(2)	(3)	(4)	(5)
	Contracted COVID-19	Sought medical intervention conditional on having an infection	Received a COVID-19 vaccine	Experienced side effects conditional on receiving a COVID-19 vaccine	Reported protecting others as a primary reason for vaccinating
Democrats	20%	25%	46%	83%	72%
Republicans	21%	24%	28%	86%	64%

Note: The table reports the share of individuals from both major U.S. political parties in the pre-experiment.

As discussed in the next section, we asked our experimental participants to bet on the COVID-19 experiences of a randomly selected participant from our pre-experiment survey. The goal is to infer experimental participants’ beliefs about how likely a participant in the pre-experiment survey is going to answer “Yes” or “No” to each question. We surveyed 100 Democrats and 100 Republicans in the pre-experiment survey. This sample size is an intuitive, easy-to-visualize natural sample size that is commonly used in the literature to help participants forming beliefs with reduced cognitive effort and improved accuracy (e.g., [63–65]).

The statistics obtained from our pre-experiment survey are in line with those available from other data sources that do not separately report results by political affiliation. First, the vaccination rate among our respondents is similar to that from the Household Pulse Survey (04/30/2024-05/27/2024) where almost 40% of respondents reported receiving a recent COVID-19 vaccine. Second, the reported rate of side effects among our respondents is also consistent with clinical trial evidence [66] and other surveys [11,67]. These similarities increase our confidence in the quality of our pre-experiment survey data.

### Experiment

We ran an experiment on Prolific to elicit participants’ beliefs on five COVID-related topics. To identify the effects of new information on people’s vaccination decision through the information shock, it is crucial to ensure an accurate measure of subjects’ initial health beliefs. To achieve this, we use incentive-compatible tasks to elicit subjects’ beliefs. Earlier empirical works indicate that beliefs elicited using incentive-compatible methods better explain the relevant decisions compared to methods with a flat payment [68–71]. Importantly, when the beliefs are politically charged in nature, people may mis-represent their beliefs to corroborate their political idealization or biases without proper incentives. For example, when asked about the belief about the vaccination rate among Republicans, a Democrat might intentionally report a lower rate than what they truly believed. Incentive compatible tasks could reduce this type of express reporting [72,73].

To achieve incentive compatibility, we ask participants to bet on the experiences collected in the pre-experiment survey, using this Binarized Scoring Rule procedure (BSR, [69,74]). The BSR is widely regarded as the state‑of‑the‑art method for incentivized belief elicitation: it delivers incentive‑compatibility under very general risk preferences while being simple to implement and empirically robust in lab comparisons [69,74,75]. Under this procedure, participant’s decisions should truthfully reveal their views on the benefits, cost, and externality value from vaccinating.

Because infections spike in the autumn and winter months [59–61], prior work has shown that many individuals receive COVID-19 vaccines in the early autumn months in conjunction with their annual influenza vaccine [76]. In June 2024, the FDA provided guidance to vaccine manufacturers about what would likely be the dominant strains during the 2024−2025 season, and the vaccines were approved 08/22/2024 [77,78]. As such, we recruited participants from 08/01/2024-08/04/2024 when participants were likely deciding whether to receive the update COVID-19 vaccine. The experiment was approved by the Institutional Review Board at University of Massachusetts Amherst under protocol #5395. All participants provide informed consent electronically. While 598 participants completed the experiment, we excluded 19 individuals whose self-reported political affiliation was inconsistent with their Prolific registration. Eleven individuals registered on Prolific as Democrats self-identified as independent (6), Republican (3), and “None of the above” (2). Eight individuals registered on Prolific as Republicans identified as independent (4), Democrat (3), and “Other” (1). The experiment was expected to take 30 minutes. All participants earned a fixed payment of $5, though participants could earn an additional $10, depending on the accuracy of their bet and a randomization process ($15 total, or a $30 hourly rate).

We informed participants that we had previously surveyed 100 self-identified Democrats/Republicans on Prolific about their COVID-19 experiences. We then asked participants to bet on whether a randomly selected respondent from our survey had answered “Yes” or “No” for each of the five questions. The participants placed their bets by allocating 100 tokens between these two outcomes, and their allocations determined the chances of receiving the additional $10 payment, depending on the randomly drawn respondent’s response. Under BSR, participants maximize their payoffs when the share of tokens allocated to “Yes” is equal to their subjective belief about the share of “Yes” responses in the survey. Participants used a slider to allocate tokens and instantly saw the calculated probability of winning the bonus payment [79,80]. S1 Fig shows an example interface. We allowed participants to familiarize themselves with this interface using a practice bet, and we emphasized that participants were best off allocating their tokens based on their true beliefs [81].

Table 2 reports summary statistics for the full sample (column 1), Democrats (column 2), and Republicans (column 3). Like prior work [26], we find that almost 38% of participants reported an intent to receive a COVID-19 vaccine. However, this figure masks partisan heterogeneity. On average, Democrats were more likely to report an intent to vaccinate than Republicans (50% vs. 25%). Consistent with our pre-experiment survey, we find that over 40% of Democrats reported previously vaccinating compared to approximately 30% of Republicans.

**Table 2:** Summary statistics for experiment participants.

	Full sample	Democrats	Republicans
**COVID-19 vaccination**			
Plans to receive a COVID-19 vaccine	0.377	0.500	0.254
	(0.485)	(0.501)	(0.436)
Has received a COVID-19 vaccine	0.359	0.413	0.305
	(0.480)	(0.493)	(0.462)
**Beliefs about others’ COVID-19 Experiences**			
Share Contracting COVID-19	0.420	0.420	0.419
	(0.244)	(0.235)	(0.252)
Share of infected with a severe infection	0.466	0.440	0.493
	(0.255)	(0.249)	(0.258)
Vaccination Rate	0.458	0.428	0.487
	(0.283)	(0.274)	(0.288)
Share with side effects from vaccination	0.506	0.546	0.467
	(0.257)	(0.251)	(0.257)
share vaccinating to protect others	0.554	0.547	0.561
	(0.264)	(0.254)	(0.274)
**Demographic characteristics**			
Age	39.58	38.04	41.10
	(13.45)	(12.68)	(14.03)
Elderly person in the household	0.126	0.125	0.127
	(0.332)	(0.331)	(0.334)
has health insurance	0.888	0.896	0.880
	(0.316)	(0.306)	(0.326)
has a primary care provider	0.758	0.750	0.766
	(0.429)	(0.434)	(0.424)
White	0.656	0.594	0.718
	(0.475)	(0.492)	(0.451)
Black	0.233	0.243	0.223
	(0.423)	(0.430	(0.417)
Other	0.111	0.163	0.058
	(0.314)	(0.370)	(0.235)
Female	0.582	0.639	0.526
	(0.494)	(0.481)	(0.500)
Male	0.409	0.344	0.474
	(0.492)	(0.476)	(0.500)
Non-binary/other gender	0.009	0.017	0.000
	(0.093)	(0.131)	(0.000)
At most a high school degree	0.119	0.111	0.127
	(0.324)	(0.315)	(0.334)
Some college	0.316	0.319	0.313
	(0.466)	(0.467)	(0.464)
College graduate	0.565	0.569	0.560
	(0.496)	(0.496)	(0.497)
Household income < $50K	0.283	0.288	0.278
	(0.451)	(0.454)	(0.449)
Household income $50K-$100K	0.392	0.417	0.368
	(0.489)	(0.494)	(0.483)
House income $100K+	0.325	0.295	0.354
	(0.469)	(0.457)	(0.479)
Observations	579	288	291

Table 3 reports the difference between participants’ beliefs and the shares from our pre-experiment survey, overall and by political concordance. S2 Fig plots the belief distributions. S1 Table reports statistics by political party. Participants overestimated the likelihood of contracting COVID-19 by 20–22 percentage points (Panel A column 1), regardless of political concordance. Likewise, participants overestimated the share of infected individuals seeking medical attention by 22 percentage points (Panel A column 2). While the errors were smallest in magnitude for vaccination rates (Panel A column 3), participants still overestimated these values by 7–11 percentage points. Meanwhile, participants generally underestimated the share of respondents who reported experiencing vaccine side effects (Panel A column 3), as well as the share reporting “protecting others” as the driver of their vaccination decision (Panel A column 4). Interestingly, S3 Fig shows that participants did not hold statistically more accurate beliefs about members of their concordant political party.

**Table 3:** Errors in participants’ initial health beliefs.

	(1)	(2)	(3)	(4)	(5)
	Contracted COVID-19	Sought medical intervention conditional on having an infection	Received a COVID-19 vaccine	Experienced side effects conditional on receiving a COVID-19 vaccine	Reported protecting others as a primary reason for vaccinating
**Panel A: Average error among experiment participants, full sample**					
Overall	21.46	22.15	8.83	−33.89	−12.56
	(24.43)	(25.40)	(26.37)	(25.62)	(25.05)
Concordant	22.15	22.34	10.60	−34.39	−11.25
	(24.30)	(25.13)	(27.36)	(26.57)	(24.91)
Discordant	20.78	21.97	7.09	−33.41	−13.85
	(24.56)	(25.70)	(25.28)	(24.68)	(25.16)
**Panel B: Average error among experiment participants, treated group**					
Overall	18.98	26.51	5.89	−35.54	−26.43
	(23.98)	(25.19)	(26.12)	(26.43)	(10.49)
Concordant	21.15	26.66	9.66	−34.16	−7.52
	(25.53)	(24.48)	(25.57)	(26.47)	(22.60)
Discordant	16.96	26.39	2.11	−37.73	−12.99
	(22.30)	(25.98)	(26.32)	(26.56)	(25.01)

Note: Panels A and B report the average difference between what the participants in our experiment predicted about the experiences of individuals with COVID-19 and the amount obtained in the pre-experiment survey. Standard deviations are reported in parentheses. Positive values indicate that on average participants overestimated a particular share, while negative values indicate that the participants underestimated the share. Panel A reports the average error when individuals in the full sample made predictions about the experiences, and Panel B reports these same figures but only among the subset of individuals who later had the actual values from the pre-experiment survey revealed to them.

After belief elicitation, a computer program randomly selected one of the five beliefs. Then, the program chose a random survey respondent and used the corresponding response (i.e., “Yes” or “No”) to the selected belief to determine payment. Participants were informed of the randomly drawn belief and response, as well as the share of respondents who answered “Yes” for that question. Participants were then asked about their COVID-19 vaccine intentions during the next 12 months. Finally, participants were informed whether they had won the additional $10. In a December 2024 one question follow-up survey, we asked participants whether they had received a COVID-19 vaccine during the prior four months. The 363 participants received $1, and 25.9% reported vaccinating. Given the high attrition rate, we follow prior work and do not treat this question as a primary outcome [26]. We report the average prediction errors based on the revealed belief in the final panel of Table 3. S4 Fig plots the distribution of these beliefs.

### Empirical approach

We estimate the relationship between the information shock and the subsequent vaccination decision with the following specification:


$$\begin{array}{c} {\text{VAX}}_{i}=\alpha +\sum_{\text{b}=\text{1}}^{\text{5}}\beta_{b}\cdot {\text{INFO}}_{ib}+{\text{X}}_{i}^{\prime }\gamma +\epsilon_{i} \end{array}$$ (1)

where the dependent variable is an indicator for whether the respondent, *i*, reported an intent to receive a COVID-19 vaccine. The independent variables of interest, INFO_*ib*_, denote the direction and size of the information shock that the respondent received regarding belief *b*. For the treated belief, the variable is the difference between the revealed value and the initial belief. For the four non-treated beliefs, the variables are zeroes.

We include a vector of pre-registered individual-level characteristics, X, to increase precision and account for incidental differences between the groups, including age and age squared, as well as indicators for race/ethnicity, gender, educational attainment, income level, whether there is an elderly individual within the household, whether the participant reported having health insurance coverage, whether the participant reported having a primary care physician, and whether the participant had previously opted to receive a COVID-19 vaccine.

Half of the participants were revealed information based on the experiences of members of their concordant political party, while the other half were revealed information based on the discordant party. We estimate the following pre-registered specification:


$$\begin{array}{c} {\text{VAX}}_{i}=\alpha +\sum_{\text{b}=\text{1}}^{\text{5}}\beta_{b}\cdot {\text{INFO FROM CONCORDANT PARTY}}_{ibs}+\sum_{\text{b}=\text{1}}^{\text{5}}\delta_{b}\cdot {\text{INFO FROM DISCORDANT PARTY}}_{ibs}+{\text{X}}_{i}\text{'}\gamma +\epsilon_{i} \end{array}$$ (2)

where the independent variables of interest denote the information shock to belief *b* based on the experiences of those in the concordant or discordant political party *s*.

Our pre-analysis plan proposed to estimate equations (1) and (2) via logistic regression. The study was pre-registered at Open Science Framework (https://osf.io/qnre7). To facilitate an easier interpretation, we present results estimated via ordinary least squares in the main manuscript. However, we report results using logistic regression in the supporting information to show that this deviation does not drive our results. This is the only instance where we have deviated from our pre-analysis plan.

## Results

**Fig 1:**
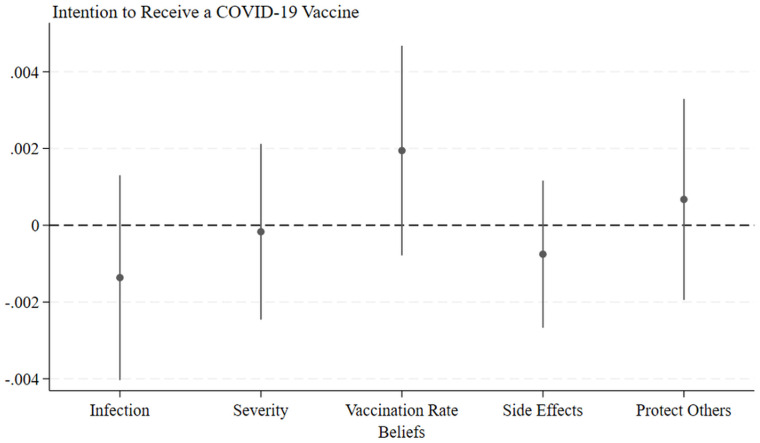
Suggestive evidence that participants who learned they underestimated the COVID-19 vaccination rate were more likely to report an intent to vaccinate. Note: The figure plots the estimates obtained from equation (1), which examines the relationship between shocks to beliefs about COVID-19 and whether individuals reported an intent to receive a COVID-19 vaccination in the next 12 months. The grey circles denote the point estimate, and the vertical lines denote the 95 percent confidence intervals.

Figure 1 explores the relationship between our information shocks and whether participants reported an intent to receive a COVID-19 vaccine. While the results are inconclusive, we find suggestive evidence that receiving a positive (negative) information shock about the COVID-19 vaccination rate was related to an increase (decrease) in the likelihood that participants reported an intent to vaccinate. S2 Table reports results using both linear and logistic regression. While not statistically significant at conventional levels (p = 0.16), the estimate implies that participants who learned that they had underestimated (overestimated) the COVID-19 vaccination rate by a standard deviation were 13% more (less) likely to report an intent to vaccinate after learning the actual vaccination rate (100 × (0.0019 × 26.12)/0.3782 = 13.12).

**Fig 2:**
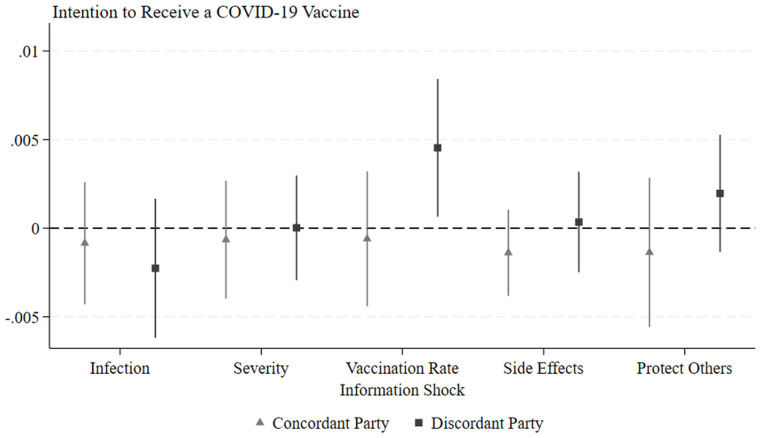
Participants who learned they underestimated the COVID-19 vaccination rate of the discordant party were more likely to report an intent to vaccinate. Note: The figure plots the estimates obtained from equation (2), which examines the importance of political concordance in shaping vaccination decision-making by separately considering whether the shocks to the individuals’ COVID-19 beliefs are based on the experiences of those from the same or opposite political party. lighter grey triangles indicate the estimates from when an individual received a shock to their beliefs based on the experiences of someone with the same political party affiliation, while the darker grey squares indicate the estimates from when an individual received a shock to their beliefs based on the experiences of someone with the opposite political party affiliation. The relationships are estimated via ordinary least squares. The vertical lines denote the 95 percent confidence intervals.

Figure 2 tests whether the information shocks were differentially salient if they were based on the experiences of members of the concordant or discordant political party. Interestingly, we find no evidence that participants’ vaccine intentions were influenced by information shocks coming from members of their concordant political party; the point estimates are small in magnitude and statistically insignificant (light grey triangles). However, we find that participants’ vaccination intentions were influenced by learning the vaccination rate among members of the discordant political party (darker grey squares). S5 Figure shows a similar pattern using logistic regression. Participants who underestimated (overestimated) the vaccination rate among members of the discordant party by one standard deviation were 31% more (less) likely to report an intent vaccinate after learning the actual vaccination rate (100 × (0.0045 × 26.32)/0.3782 = 31.32). This pattern is consistent with evidence that individuals are more open to vaccinating when they learn that others are willing to accept the vaccine [82]. S6 Figure suggests that participants who learned they underestimated the severity rate were subsequently more likely to report vaccinating, though these results are sensitive to attrition adjustments [26]. S7 Figure shows that Democrats who learned they underestimated the share of Republicans vaccinated against COVID-19 were more likely to report an intent to vaccinate. We do not find evidence of statistically significant responses among Republicans. However, S8 Fig shows suggestive evidence that previously vaccinated Republicans were responsive to learning about the share of vaccinated Democrats.

**Fig 3:**
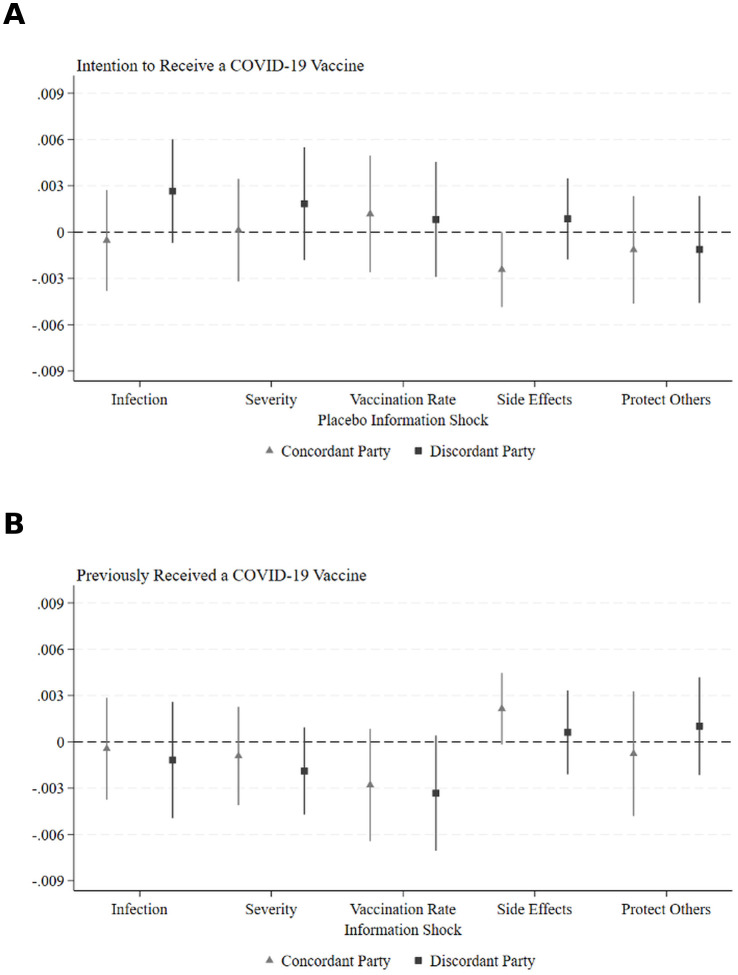
The placebo information shocks were unrelated to future vaccine intentions and the actual information shocks were unrelated to past vaccine decisions. **(A)** Placebo test. **(B)** Falsification test. Note: Panel A reports results from a placebo exercise whereby participants are assumed to have been exposed to one of the other four randomly selected information shocks. Panel B reports results examining the relationship between the actual information shocks and the likelihood that participants reported previously receiving a COVID-19 vaccine. The relationships are estimated via ordinary least squares. The estimates are obtained from equation (2), which examines the importance of political concordance in shaping vaccination decision-making by separately considering whether the shocks to the individuals’ COVID-19 beliefs are based on the experiences of those from the concordant or discordant political party. The lighter grey triangles indicate the estimates from when an individual received a shock to their beliefs based on the experiences of someone with the same political party affiliation, while the darker grey squares indicate the estimates from when an individual received a shock to their beliefs based on the experiences of someone with the opposite political party affiliation. The vertical lines denote the 95 percent confidence intervals.

Figure 3 reports results from two exercises designed to increase confidence that the estimated relationship is being driven by the information shock and not simply a spurious relationship. First, for each participant we randomly selected one of the non-treated beliefs and took the difference between the unrevealed share from our pre-experiment survey and the participant’s initial belief. Reassuringly, there is no evidence that any of these placebo shocks were related to changes in vaccination intent (Panel A). This assumes participants did not update their non-treated beliefs in response to the realized information shock [83,84]. Second, we replaced our dependent variable with an indicator for whether the participant reported previously receiving a COVID-19 vaccine. Reassuringly, the point estimates from this falsification test are smaller in magnitude, opposite signed, and statistically insignificant (Panel B).

**Fig 4:**
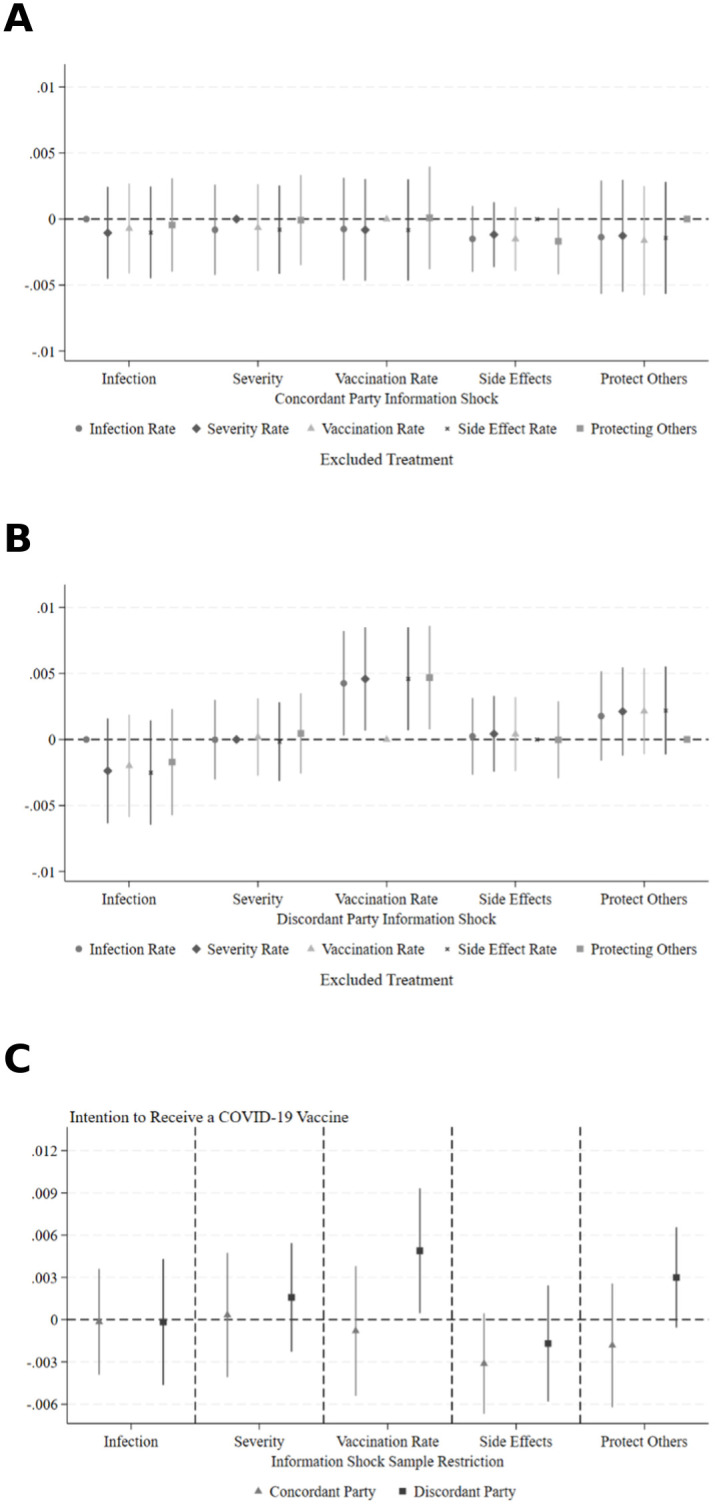
The pattern is robust to iteratively dropping participants who received each information shock and limiting comparisons to participants receiving the same category of shock. **(A)** Concordant party info. Shock estimates. **(B)** Discordant party info. Shock estimates. **(C)** Sample restriction estimates. Note: Panels A and B plot results obtained from estimating the single regression specification, shown in equation (2), five times. The circles denote a regression excluding observations that received first treatment, the diamonds denote a regression excluding observations that received the second treatment, the triangles denote a regression excluding observations that received the third treatment, the Xs denote a regression excluding observations that received the fourth treatment, and the squares denote a regression excluding observations that received the fifth treatment. Panel A reports the estimates for the concordant party shocks, while Panel B reports the estimates for the discordant party shocks. Panel C plots the estimates obtained from five separate regressions using equation (2). In each regression, the sample is limited to individuals who received the same health belief information shock. The lighter grey triangles indicate the estimates from when an individual received a shock to their beliefs based on the experiences of someone with the same political party affiliation, while the darker grey squares indicate the estimates from when an individual received a shock to their beliefs based on the experiences of someone with the opposite political party affiliation. The relationships are all estimated via ordinary least squares. The vertical lines denote the 95 percent confidence intervals.

Figure 4 further explores the sensitivity of our results. First, we show that the patterns are robust to iteratively excluding participants who received each category of information shock (Panels A and B). Second, we show that the results are robust to limiting the sample to participants receiving the same category of information shock (Panel C). This latter restriction leverages variation in the size of the information shock relative to participants’ initial beliefs.

## Conclusion

Over the last decade, researchers have become increasingly interested in understanding the economic and policy determinants of vaccine decision-making. This work has shown that individuals are more likely to get vaccinated when they are aware of the benefits of vaccination, either due to increased salience generated by disease outbreaks [40,85,43] or government recommendations [39,86]. Meanwhile, other work has shown that individuals are influenced by the vaccine experiences of those in their households and social networks [41,87] and those of similar racial and educational backgrounds [26]. Given the growing evidence that that political identity shapes how individuals acquire and process information [88–91], it is possible that political identity likewise affects the salience of vaccine-related information.

In this paper, we explore the role of political identity in COVID-19 vaccination. Regardless of political affiliation, we find that participants overestimated the frequency and severity of COVID-19 infections among members of both major political parties. We consistently find that participants who learned that they had underestimated (overestimated) the vaccination rate among members of the discordant political party were more (less) likely to report an intent vaccinate. This pattern is consistent with a social pressure model where individuals are more inclined to get vaccinated when they learn that others have done the same [82]. In contrast, we do not find any evidence that participants were more or less likely to report an intention to receive a COVID-19 vaccine when presented with updated information about the COVID-19 infection and severity rates, the vaccine side effect rate, or the share of individuals who reported vaccinating in an effort to protect others.

What do our results imply about how public health officials should go about designing information campaigns? Our results indicate that people are more inclined to vaccinate when they learn that others have received the vaccine and less inclined to vaccinate when they learned that others have not. Because most participants overestimated COVID-19 vaccination rates, our results suggest that efforts to inform the public about low vaccine take-up may inadvertently discourage vaccination. Prior research has shown that information campaigns highlighting that many people are engaging in socially undesirable behaviors may inadvertently undercut efforts to discourage those behaviors [92]. For example, individuals who learned that they were conserving more energy than their neighbors subsequently reduced their conservation efforts [93]. In this case, messaging emphasizing the socially optimal vaccination rate is likely to be more effective than messaging reporting the existing vaccination rate [92].

This study is subject to some limitations. For one, sample size constraints prevented us from performing granular heterogeneity exercises. Additionally, our study does not allow us to say why participants were more responsive to learning the vaccination rate of members of the discordant political party relative to the concordant party. Future studies should consider methods for disentangling the potential mechanisms. Despite these limitations, our study provides important new evidence on how political identity shapes individuals’ responses to health information.

## Supporting information

S1 Text Pre-experiment survey. The full text of the pre-experiment survey instrument.
(DOCX)

S1 Fig Bet infographic. The figure shows the infographic displayed to participants that allowed them to see the relationship between their token allocation and the likelihood that would win if a randomly drawn respondent had answered “yes” or “no” to the relevant question.
(TIF)

S2 Fig Distribution of beliefs among the full sample. The panels plot the distributions of beliefs for the full sample. The solid black line denotes the value obtained from our pre-experiment survey. Panel A plots the predicted share who contracted COVID-19, Panel B plots the predicted share who required medical intervention conditional on contracting COVID-19, Panel C plots the share who received a COVID-19 vaccine, Panel D plots the predicted share of individuals experiencing side effects conditional on receiving a COVID-19 vaccine, and Panel E plots the share of individuals who report “protecting others” as a primary reason that they opted to receive the vaccine.
(TIF)

S3 Fig P-Values showing that participants did not have more accurate beliefs about the COVID-19 experiences among their own political party. The figure plots the p-values obtained from Kolmogorov-Smirnov Equality-of-Distribution tests examining whether participants were differentially likely to predict the COVID-19 experiences of members of the same or opposite political party.
(TIF)

S4 Fig Distribution of beliefs among treated individuals. The panels plot the belief distributions among those receiving shocks to those beliefs. The solid black line denotes the value obtained from our pre-experiment survey. Panel A plots the predicted share who contracted COVID-19, Panel B plots the predicted share who required medical intervention conditional on contracting COVID-19, Panel C plots the share who received a COVID-19 vaccine, Panel D plots the predicted share of individuals experiencing side effects conditional on receiving a COVID-19 vaccine, and Panel E plots the share of individuals who report “protecting others” as a primary reason that they opted to receive the vaccine.
(TIF)

S5 Fig The pattern is robust to using logistic regression. The figure plots the estimates obtained from equation (2), which examines the importance of political concordance in shaping vaccination decision-making by separately considering whether the shocks to the individuals’ COVID-19 beliefs are based on the experiences of those from the same or opposite political party. The specification is estimated using logistic regression and the coefficients are the log odds ratios. The lighter grey triangles indicate the estimates from when an individual received a shock to their beliefs based on the experiences of someone with the same political party affiliation, while the darker grey squares indicate the estimates from when an individual received a shock to their beliefs based on the experiences of someone with the opposite political party affiliation. The vertical lines denote the 95 percent confidence intervals.
(TIF)

S6 Fig Participants who learned they underestimated the COVID-19 severity rate were more likely to report that they received a COVID-19 vaccine in the follow-up survey. The dependent variable is an indicator for whether the participant reported having received a COVID-19 vaccine during the prior four months when they completed the survey in December 2024. The figures plot estimates obtained from equation (2), which examines the importance of political concordance in shaping vaccination decision-making by separately considering whether the shocks to the individuals’ COVID-19 beliefs are based on the experiences of those from the same or opposite political party. The relationship is estimated via ordinary least squares. The lighter grey triangles indicate the estimates from when an individual received a shock to their beliefs based on the experiences of someone with the same political party affiliation, while the darker grey squares indicate the estimates from when an individual received a shock to their beliefs based on the experiences of someone with the opposite political party affiliation. The vertical lines denote the 95 percent confidence intervals. Panel A only uses data for the 363 participants who responded to the follow-up survey. Panel B uses the full data and treats non-respondents as unvaccinated (Alsan and Eichmeyer 2024). Panel C uses the full data and treats non-respondents as vaccinated.
(TIF)

S7 Fig Democrats were more likely to report an intent to receive a COVID-19 vaccine after learning the COVID-19 vaccination rate among republicans. Panels A and B plots estimates obtained from a single regression whereby we interact our independent variables of interest with an indicator for each political party. The relationship is estimated via ordinary least squares. Panel A plots the estimates for Democrats while Panel B plots the estimates for Republicans. The darker grey triangles indicate the estimates from when an individual received a shock to their beliefs based on the experiences of someone with the same political party affiliation, while the lighter grey squares indicate the estimates from when an individual received a shock to their beliefs based on the experiences of someone with the opposite political party affiliation. The vertical lines denote the 95 percent confidence intervals.
(TIF)

S8 Fig There is suggestive evidence that participants from both parties who were previously vaccinated were more likely to report an intent to receive a COVID-19 vaccine after learning the COVID-19 vaccination rate among members of the discordant political party. The figure plots estimates obtained from a specification our independent variables of interest in equation (2) with indicators for whether the participant was a Democrat or Republican and whether the participant reported previously receiving a COVID-19 vaccine. For brevity, we only report the estimates on the information shock of interest which measures the difference between participants’ initial beliefs and the revealed vaccination rate among members of the concordant or discordant political party. The estimates are obtained via ordinary least squares. The lighter grey triangles indicate the estimates from when an individual received a shock to their beliefs based on the experiences of someone with the same political party affiliation, while the darker grey squares indicate the estimates from when an individual received a shock to their beliefs based on the experiences of someone with the opposite political party affiliation. The vertical lines denote the 95 percent confidence intervals.
(TIF)

S1 Table Average errors in participants’ beliefs about the experiences of democrats and republicans with COVID-19 during the past 12 months. Each panel reports the average difference between what the participants in our experiment predicted about the experiences of individuals with COVID-19 and the amount obtained in the pre-experiment survey. Standard deviations are reported in parentheses. Positive values indicate that on average participants overestimated a particular share, while negative values indicate that the participants underestimated the share. Panel A reports the average error when individuals in the full sample made predictions about the experiences of Democrats, and Panel B reports the average error when individuals in the full sample made predictions about Republicans. Panels C and D report these same figures but only among the subset of individuals who later had the actual values from the pre-experiment survey revealed to them.
(DOCX)

S2 Table Baseline relationship when not accounting for political concordance. The table reports the estimates obtained from equation (1), which examines the relationship between shocks to beliefs about COVID-19 and whether individuals reported that they intend to receive a COVID-19 vaccination in the next 12 months. Column 1 is estimated via ordinary least squares, while column 2 is estimated via logistic regression and reports the log odds ratios.
(DOCX)
