# Supplementary material for: Partisan differences in healthcare decision-making: Evidence from a vaccine experiment
Source: PLoS One. 2026 Jul 20;21(7):e0352319. doi: 10.1371/journal.pone.0352319 (PMC13384293; doi:10.1371/journal.pone.0352319)
Supplement: S1 Text — The full text of the pre-experiment survey instrument. (DOCX) [file pone.0352319.s001.docx]

## S1 Text. Pre-experiment survey

**Online Survey Consent Form**
 You are being invited to participate in a research study titled “Health Experience Survey”. This study is being done by Dr. Sherry Gao, Dr. Brandyn Churchill, and Dr. Rong Rong from the University of Massachusetts Amherst. You were selected to participate in this study because you are an active member of the Prolific.com community.

 **Why are we doing this research study?**
 The purpose of this research study is people’s health-related experiences in the past 12 months. Who can participate in this research study? Anybody who is at least 18 years old and is part of the Prolific.com community can participate in this survey.

 **What will I be asked to do and how much time will it take?**
 If you agree to take part in this study, you will be asked to complete an online survey. This survey will ask about your health-related experience in the past 12 months, and it will take you approximately 10 minutes to complete.

 **Will being in this research study help me in any way?**
 You may not directly benefit from this research; however, we hope that your participation in the study may help us advance our research goal.

 **What are my risks of being in this research study?**
 We believe there are minimal risks associated with this research study; however, a risk of breach of confidentiality always exists and we have taken the steps to minimize this risk as outlined in a section below.

 **How will my personal information be protected?**
 To the best of our ability your answers in this study will remain confidential. We will minimize any risks by removing your Prolific ID from the dataset once we have confirmed all payments.

 **Will I be given any money or other compensation for being in this research study?**
 You will receive $3 for completing this survey. If you choose to exit the survey before its competition, you forfeit the payment entirely and we will have access to the data you provided up until the point of your exiting.

 **What happens if I say yes, but I change my mind later?**
 You do not have to be in this study if you do not want to. If you agree to be in the study, but later change your mind, you may drop out at any time. There are no penalties or consequences of any kind if you decide that you do not want to participate.

 **Who can I talk to if I have questions?**
 If you have questions about this project or if you have a research-related problem, you may contact the researcher(s), Dr. Sherry Gao (sherrygao@resecon.umass.edu) or Dr. Rong Rong (rrong@umass.edu). If you have any questions concerning your rights as a research subject, you may contact the University of Massachusetts Amherst Human Research Protection Office (HRPO) at (413) 545-3428 or humansubjects@ora.umass.edu.

 By clicking “I agree” below you are indicating that you are at least 18 years old, have read this consent form and agree to participate in this research study. You are free to skip any question that you choose. You may print a copy of this page for your records.

- I agree. (1)
- I do not wish to participate. (2)

Thank you for considering participating in today's survey. Please enter your Prolific ID. The study is conducted by researchers at the University of Massachusetts Amherst. The study is approved by the university’s Institutional Review Board and complies with the ethical conduct required in research of this type (Protocol ID #5397). You will be compensated $3 for completing today's survey. To receive this payment, please make sure you get to the last page and get the completion code. You will need to enter the completion code back at Prolific to get paid.

**Please enter your Prolific ID below. Please make sure you enter it correctly so that you can receive the payment for participating in this survey on Prolific.**

Q1 Have you received a COVID-19 vaccine (initial shot or booster) in the past 12 months?

- No (1)
- Yes (2)

Q5 Why did you choose to not receive a COVID vaccine in the past 12 months? For each of the reasons listed below, please indicate how important the reason is to your decision.

|  | Not at all important (9) | Slightly important (10) | Moderately important (11) | Very important (12) | Extremely important (13) |
| --- | --- | --- | --- | --- | --- |
| I was not recommended the vaccine by a healthcare professional (1) |  |  |  |  |  |
| I do not believe that the vaccine is medically necessary (2) |  |  |  |  |  |
| I am worried about the vaccine’s safety or side effects (3) |  |  |  |  |  |
| The vaccine costs too much money (4) |  |  |  |  |  |
| It is difficult for me to schedule and travel to a vaccine appointment (5) |  |  |  |  |  |
| I am already fully vaccinated (6) |  |  |  |  |  |
| I have natural immunity (7) |  |  |  |  |  |
| It is against my religious and/or philosophical beliefs (8) |  |  |  |  |  |
| I believe the vaccine is not effective (9) |  |  |  |  |  |
| Another reason that is not mentioned above (10) |  |  |  |  |  |

Q6 You said that you did not receive a COVID vaccine during the past 12 months. Have you **ever** received a COVID vaccine?

- No (1)
- Yes (2)

Q34 Why did you choose to receive a COVID vaccine in the past 12 months? For each of the reasons listed below, please indicate how important the reason is to your vaccination decision. 

|  | Not at all important (15) | Slightly important (16) | Moderately important (17) | Very important (18) | Extremely important (19) |
| --- | --- | --- | --- | --- | --- |
| I believe that the COVID vaccine will reduce my **chance of infection**. (1) |  |  |  |  |  |
| I believe that the COVID vaccine will reduce **my symptoms if I become infected**. (2) |  |  |  |  |  |
| I believe that by getting the COVID vaccine I will reduce the chance other people **in my household** become infected. (3) |  |  |  |  |  |
| I believe that by getting the COVID vaccine I will reduce the chance other people **not in my household** become infected. (4) |  |  |  |  |  |
| The vaccine is free for me. (5) |  |  |  |  |  |
| My doctor recommended it to me. (6) |  |  |  |  |  |
| It is recommended or required by my employer or my school. (7) |  |  |  |  |  |
| Another reason that is not mentioned above (8) |  |  |  |  |  |

Q3 Did you experience any of the following side effects from your **most recent** COVID vaccine?
 **Please check all that apply:**

- Pain, swelling, or redness where the shot is given (1)
- Fever or chills (2)
- Tiredness (fatigue) (3)
- Headache (4)
- Muscle pain (5)
- joint pain (11)
- Nausea, vomiting (6)
- Swollen lymph nodes (7)
- Myocarditis (inflammation of the heart muscle) or pericarditis (inflammation of the lining outside the heart) (8)
- Another side effect that is not listed above (9) __________________________________________________
- None of the above (10)

Q4 How many COVID vaccines (including boosters) have you received in total since the first COVID vaccine became available in 2020?

Q7 Have you contracted COVID in the past 12 months?

- No (1)
- Yes (2)

Q31 For your **most recent** COVID infection, did you contract it before or after receiving your **most recent** COVID vaccine?

- Before vaccination (1)
- After vaccination (2)

Q8 For your **most recent** COVID infection, did you receive any of the following positive diagnoses?

**Please check all that apply.**

- Positive antigen test self-administered at home (1)
- Positive PCR or antigen test administered by a healthcare provider (2)
- Positive diagnosis from a healthcare provider without a test (3)
- None of the above (4)

Q9 For your **most recent** COVID infection, did you experience any of the following symptoms? Please check all that apply.

- Fever or chills (1)
- Sore throat, congestion or runny nose (2)
- Cough (3)
- Shortness of breath or difficulty breathing (4)
- Fatigue (tiredness) (5)
- Muscle or body aches (6)
- Headache (7)
- New loss of taste or smell (8)
- Nausea or vomiting (9)
- Diarrhea (10)
- Persistent pain or pressure in the chest (11)
- Confusion (12)
- Inability to wake or stay awake (13)
- Pale, gray, or blue‑colored skin, lips, or nail beds. (14)
- Another symptom that is not listed above (15) __________________________________________________

Q10 For your **most recent** COVID infection, did you visit a physician to address the symptoms?

- No (1)
- Yes (2)

Q11 For your **most recent** COVID infection, did you receive an anti-viral prescription (such as Paxlovid, Veklury, and Lageviro) to treat the infection?

- No (1)
- Yes (2)

Q14 For your **most recent** COVID infection, how long did it take for you to fully recover from the infection (i.e. free of all symptoms)?

- 0 day (It was asymptomatic.) (1)
- 1-3 days (2)
- 4-7 days (3)
- 8-14 days (4)
- 14-30 days (5)
- More than 30 days (6)

Q15 For your **most recent** COVID infection, how many days of work/school did you miss because of this COVID infection?

- 0 days (I did not miss any work or school) (1)
- 1-3 days (2)
- 4-7 days (3)
- 8-14 days (4)
- 14-30 days (5)
- More than 30 days (6)
- I am not employed or in school (7)

Q16 In general, what is your political affiliation?

- Democrat (1)
- Republican (2)
- Independent (3)
- Other (4) __________________________________________________
- None of the above (5)

Q33 On a scale of 1 to 7, please choose your position on the political spectrum below.

|  | Core Liberal | Lean Democrat | Neutral | Lean Republican | Core Republican | I am not political. |
| --- | --- | --- | --- | --- | --- | --- |

|  | 1 | 2 | 3 | 4 | 5 | 6 | 7 |
| --- | --- | --- | --- | --- | --- | --- | --- |

| Your Political Position () | 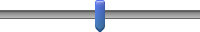 |
| --- | --- |

Q17 Which year were you born?

________________________________________________________________

Q37 What is your ethnicity?

- Hispanic (2)
- Non-Hispanic (3)

Q18 What is your race?

- White or Caucasian (1)
- Black or African American (2)
- Asian or Pacific Islander (4)
- American Indian or Alaska Native (3)
- Multiracial (8)
- Other (6) __________________________________________________

Q19 What is the gender you identify with?

- Male (1)
- Female (2)
- Non-binary / third gender (3)
- Prefer not to say (4)

Q20 What is the highest education level you have attained?

- Less than high school graduate (1)
- High school graduate or GED (2)
- Some college or associate degree (3)
- Bachelor's degree (5)
- Master's Degree (7)
- Doctoral Degree (8)

Q22 What is your current employment status?

- Employed full time (1)
- Employed part time (2)
- Unemployed looking for work (3)
- Unemployed not looking for work (4)
- Retired (5)
- Full time student (6)
- Disabled (7)

Q21 What is the range of your annual pre-tax household income?

- Less than $10,000 (1)
- $10,000 - $19,999 (2)
- $20,000 - $29,999 (3)
- $30,000 - $39,999 (4)
- $40,000 - $49,999 (5)
- $50,000 - $59,999 (6)
- $60,000 - $69,999 (7)
- $70,000 - $79,999 (8)
- $80,000 - $89,999 (9)
- $90,000 - $99,999 (10)
- $100,000 - $149,999 (11)
- More than $150,000 (12)

Q23 How many people currently reside in your household?

________________________________________________________________

Q24 What is the age of the **oldest** member of your household who resides with you?

- In Years (1) __________________________________________________

Q26 What is the age of the **youngest** member of your household who resides with you?

- In Years (1) __________________________________________________

Q28 Do you currently have health insurance?

- No (1)
- Yes (3)
- I am not sure. (4)

Q30 Do you have a medical professional you see for routine visits and checkups, often referred to as a Primary Care Provider (PCP)?

- No (1)
- Yes (2)

Q27 Choose the option that best reflects your current physical health status.

|  | Poor (1) | Fair (2) | Good (3) | Very Good (4) | Excellent (5) |
| --- | --- | --- | --- | --- | --- |
| I would describe my physical health as... (1) |  |  |  |  |  |

Q35 Have you experienced any of the following health conditions in the past 12 months?

**Please check all that apply.**

- Pregnancy (1)
- Chronic kidney, liver, or lung disease (2)
- Obesity, overweight, or physically inactive (3)
- Smoker (current or ever in your lifetime) (4)
- Substance use disorder (such as alcohol, opioid, or cocaine use disorder) (5)
- Diabetes (type 1 or type 2) (6)
- Cancer (currently or ever in your lifetime) (7)
- Heart failure, coronary artery disease, high blood pressure, or cardiomyopathies (8)
- Diseases that cause weakened immune systems, including HIV (9)
- Solid organ or blood stem cell transplant (10)
- Stroke or cerebrovascular disease (11)
- Cystic fibrosis (12)
- Sickle cell disease or thalassemia (13)
- Dementia or Alzheimer's disease (14)
- Tuberculosis (15)
- Depression, depression, and schizophrenia spectrum disorders, or other mood disorders (16)
- Disabilities (e.g., ADHD, cerebral palsy, birth defects, learning disabilities, spinal cord injuries, Down syndrome) (17)
