# Supplementary figures and images for: Partisan differences in healthcare decision-making: Evidence from a vaccine experiment
Source: PLoS One. 2026 Jul 20;21(7):e0352319. doi: 10.1371/journal.pone.0352319 (PMC13384293; doi:10.1371/journal.pone.0352319)

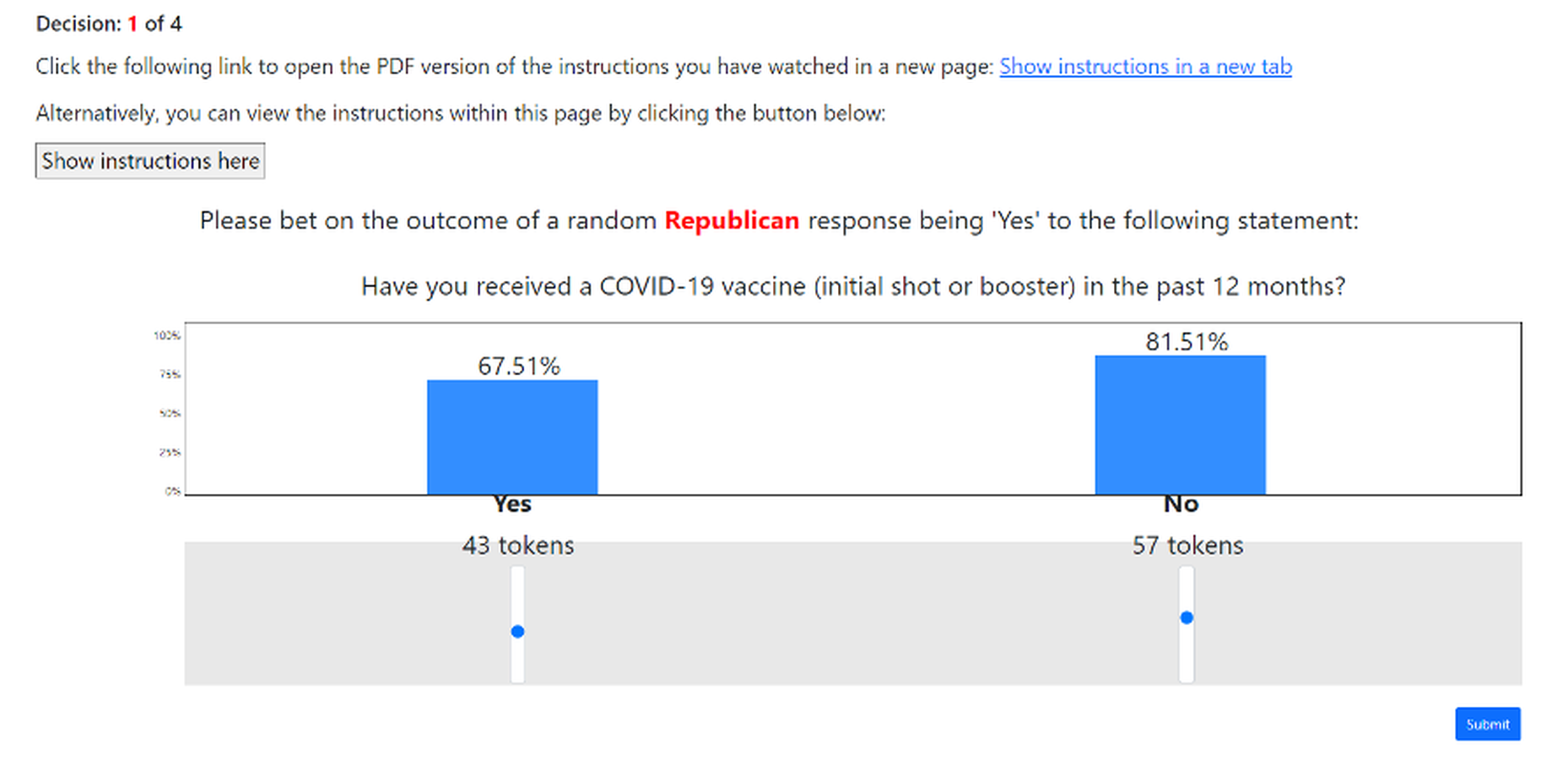

Supplement: S1 Fig — The figure shows the infographic displayed to participants that allowed them to see the relationship between their token allocation and the likelihood that would win if a randomly drawn respondent had answered “yes” or “no” to the relevant question. (TIF) [file pone.0352319.s002.tif]

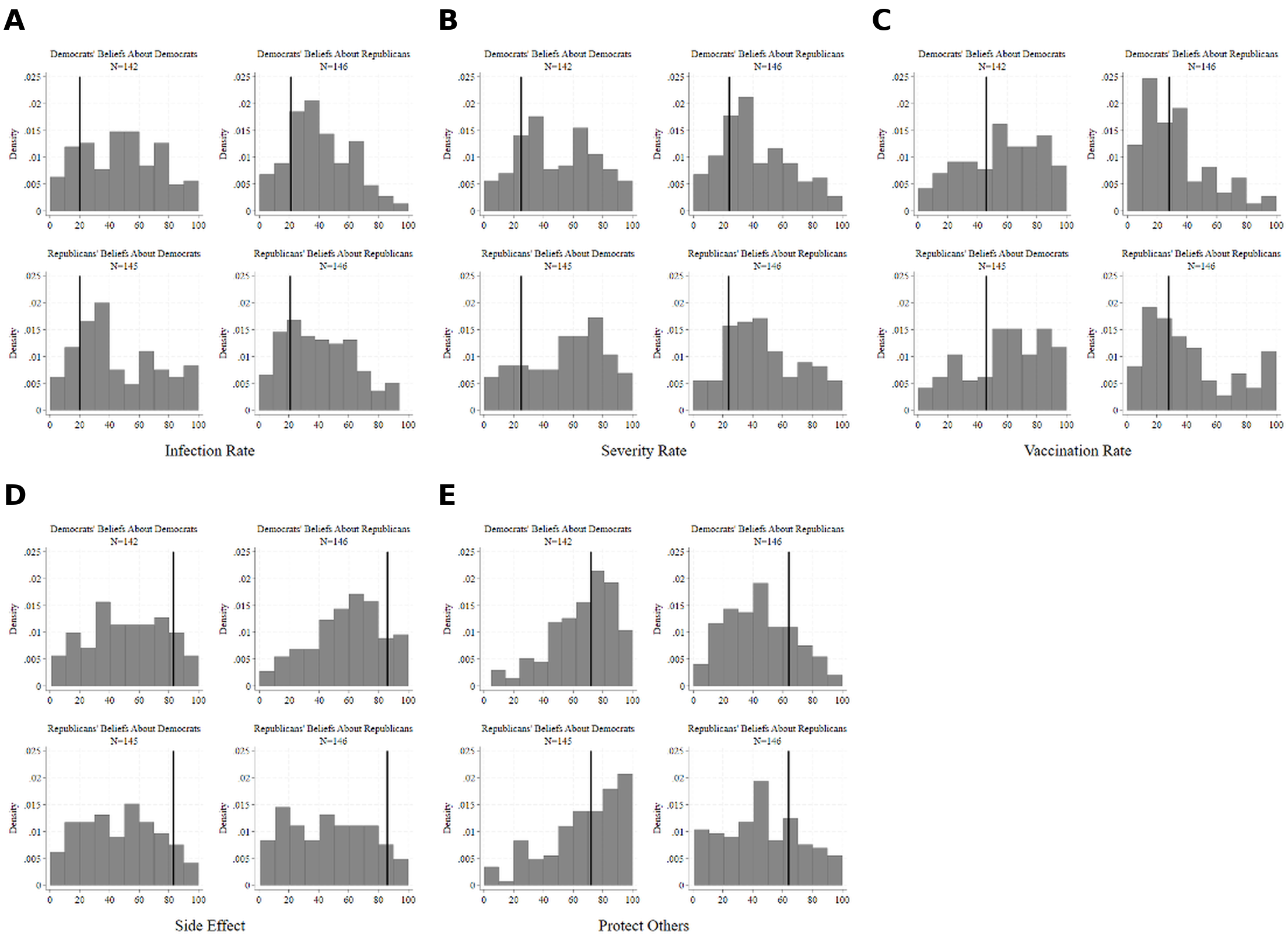

Supplement: S2 Fig — The panels plot the distributions of beliefs for the full sample. The solid black line denotes the value obtained from our pre-experiment survey. Panel A plots the predicted share who contracted COVID-19, Panel B plots the predicted share who required medical intervention conditional on contracting COVID-19, Panel C plots the share who received a COVID-19 vaccine, Panel D plots the predicted share of individuals experiencing side effects conditional on receiving a COVID-19 vaccine, and Panel E plots the share of individuals who report “protecting others” as a primary reason that they opted to receive the vaccine. (TIF) [file pone.0352319.s003.tif]

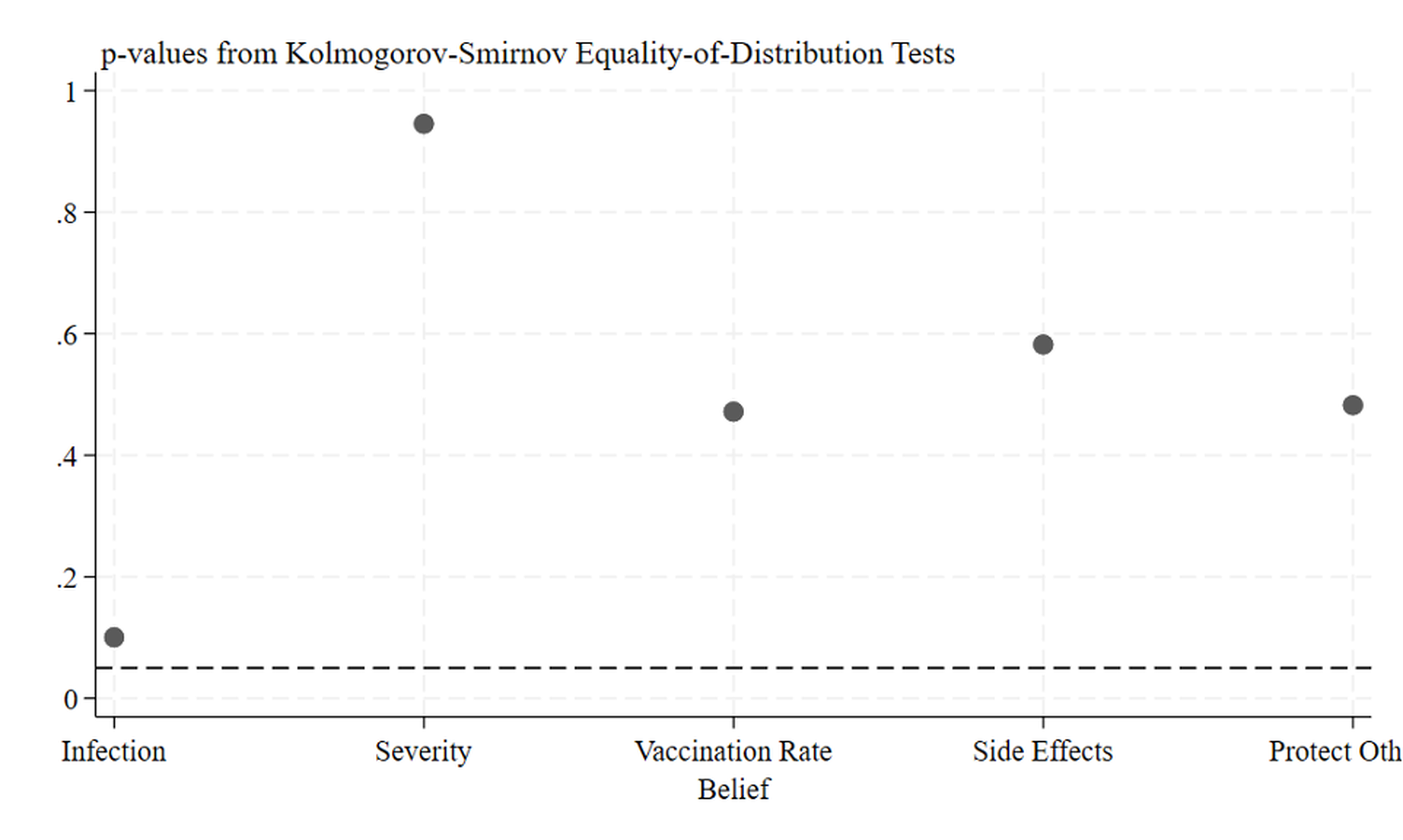

Supplement: S3 Fig — The figure plots the p-values obtained from Kolmogorov-Smirnov Equality-of-Distribution tests examining whether participants were differentially likely to predict the COVID-19 experiences of members of the same or opposite political party. (TIF) [file pone.0352319.s004.tif]

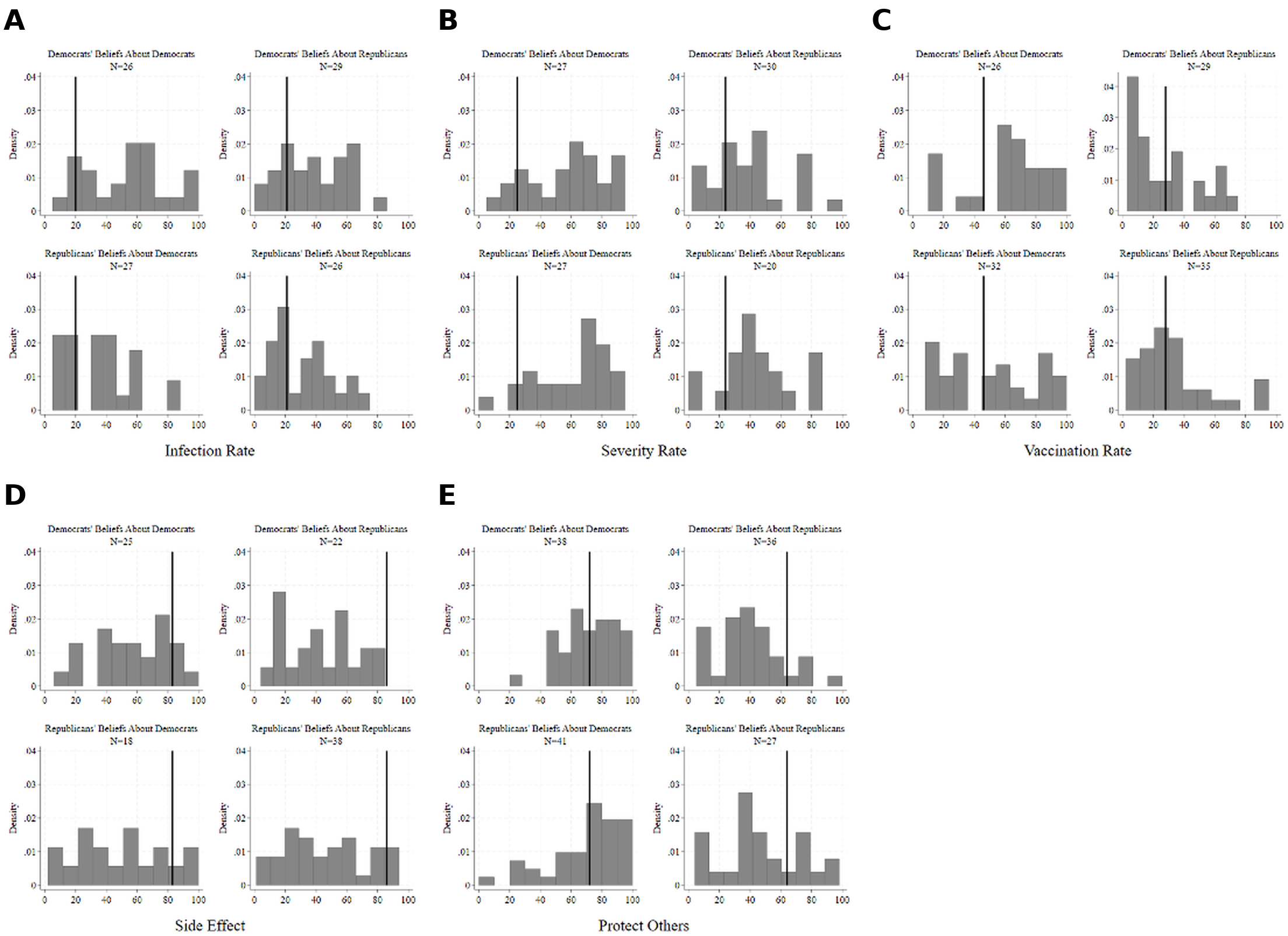

Supplement: S4 Fig — The panels plot the belief distributions among those receiving shocks to those beliefs. The solid black line denotes the value obtained from our pre-experiment survey. Panel A plots the predicted share who contracted COVID-19, Panel B plots the predicted share who required medical intervention conditional on contracting COVID-19, Panel C plots the share who received a COVID-19 vaccine, Panel D plots the predicted share of individuals experiencing side effects conditional on receiving a COVID-19 vaccine, and Panel E plots the share of individuals who report “protecting others” as a primary reason that they opted to receive the vaccine. (TIF) [file pone.0352319.s005.tif]

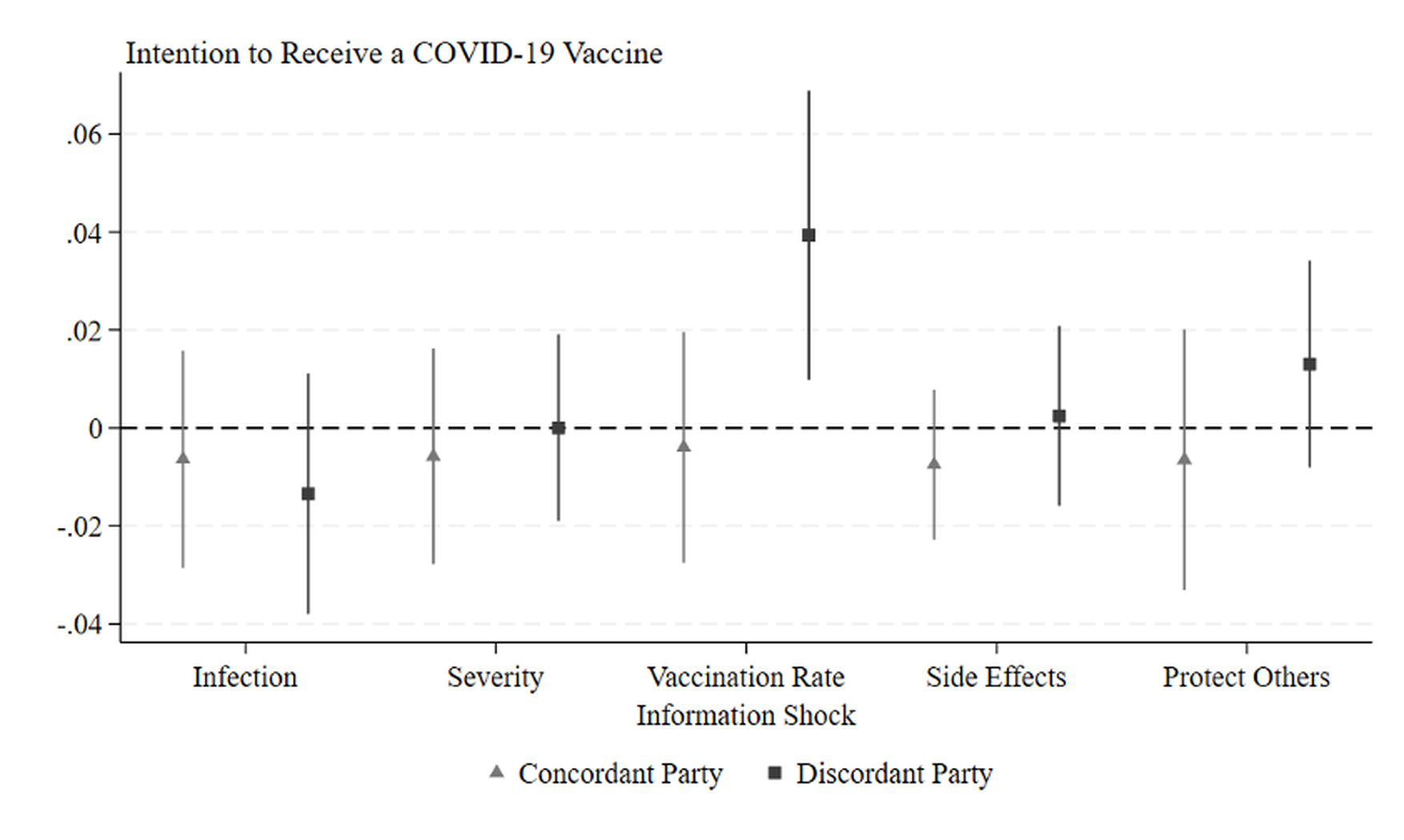

Supplement: S5 Fig — The figure plots the estimates obtained from equation (2), which examines the importance of political concordance in shaping vaccination decision-making by separately considering whether the shocks to the individuals’ COVID-19 beliefs are based on the experiences of those from the same or opposite political party. The specification is estimated using logistic regression and the coefficients are the log odds ratios. The lighter grey triangles indicate the estimates from when an individual received a shock to their beliefs based on the experiences of someone with the same political party affiliation, while the darker grey squares indicate the estimates from when an individual received a shock to their beliefs based on the experiences of someone with the opposite political party affiliation. The vertical lines denote the 95 percent confidence intervals. (TIF) [file pone.0352319.s006.tif]

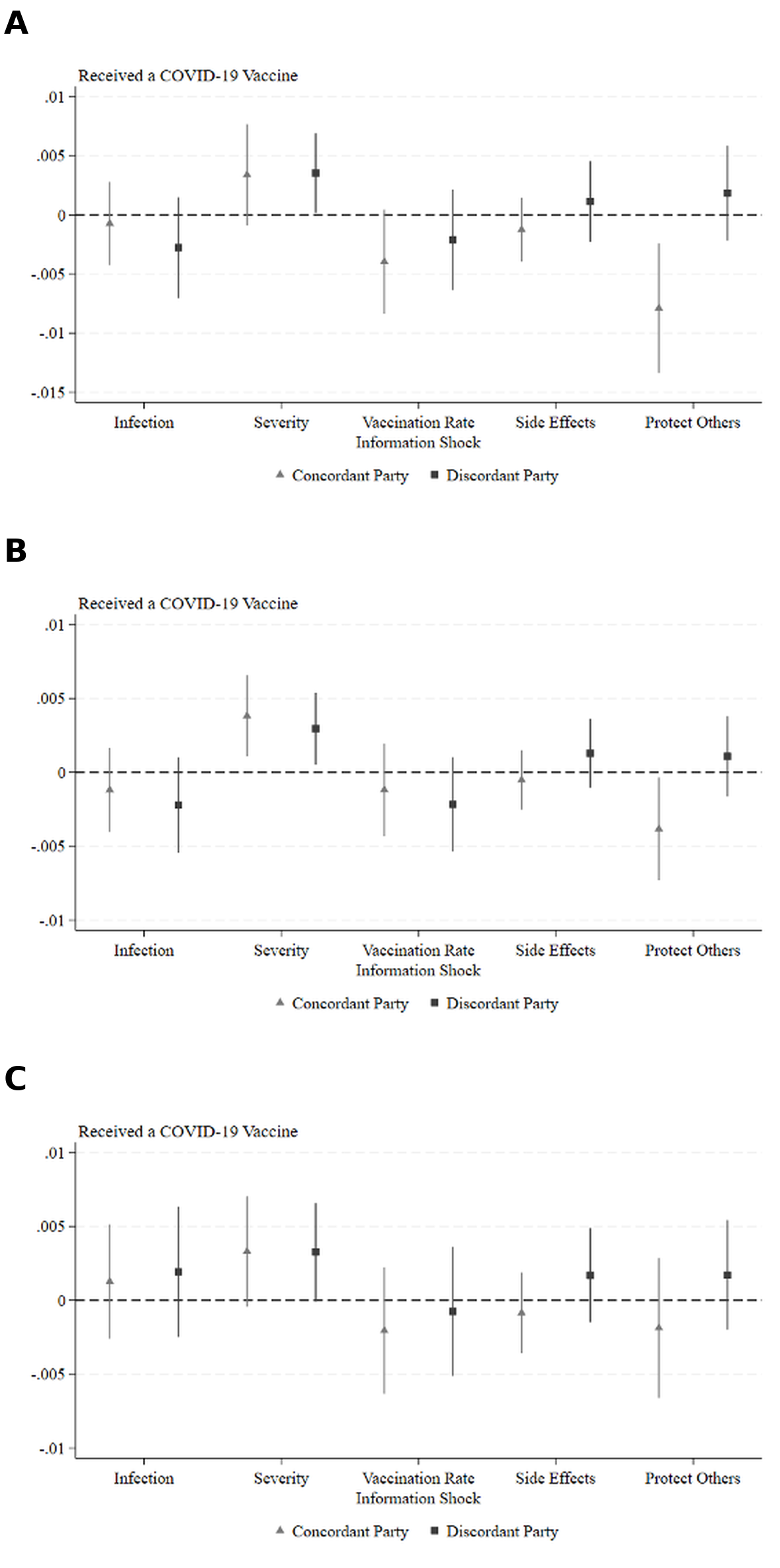

Supplement: S6 Fig — The dependent variable is an indicator for whether the participant reported having received a COVID-19 vaccine during the prior four months when they completed the survey in December 2024. The figures plot estimates obtained from equation (2), which examines the importance of political concordance in shaping vaccination decision-making by separately considering whether the shocks to the individuals’ COVID-19 beliefs are based on the experiences of those from the same or opposite political party. The relationship is estimated via ordinary least squares. The lighter grey triangles indicate the estimates from when an individual received a shock to their beliefs based on the experiences of someone with the same political party affiliation, while the darker grey squares indicate the estimates from when an individual received a shock to their beliefs based on the experiences of someone with the opposite political party affiliation. The vertical lines denote the 95 percent confidence intervals. Panel A only uses data for the 363 participants who responded to the follow-up survey. Panel B uses the full data and treats non-respondents as unvaccinated (Alsan and Eichmeyer 2024). Panel C uses the full data and treats non-respondents as vaccinated. (TIF) [file pone.0352319.s007.tif]

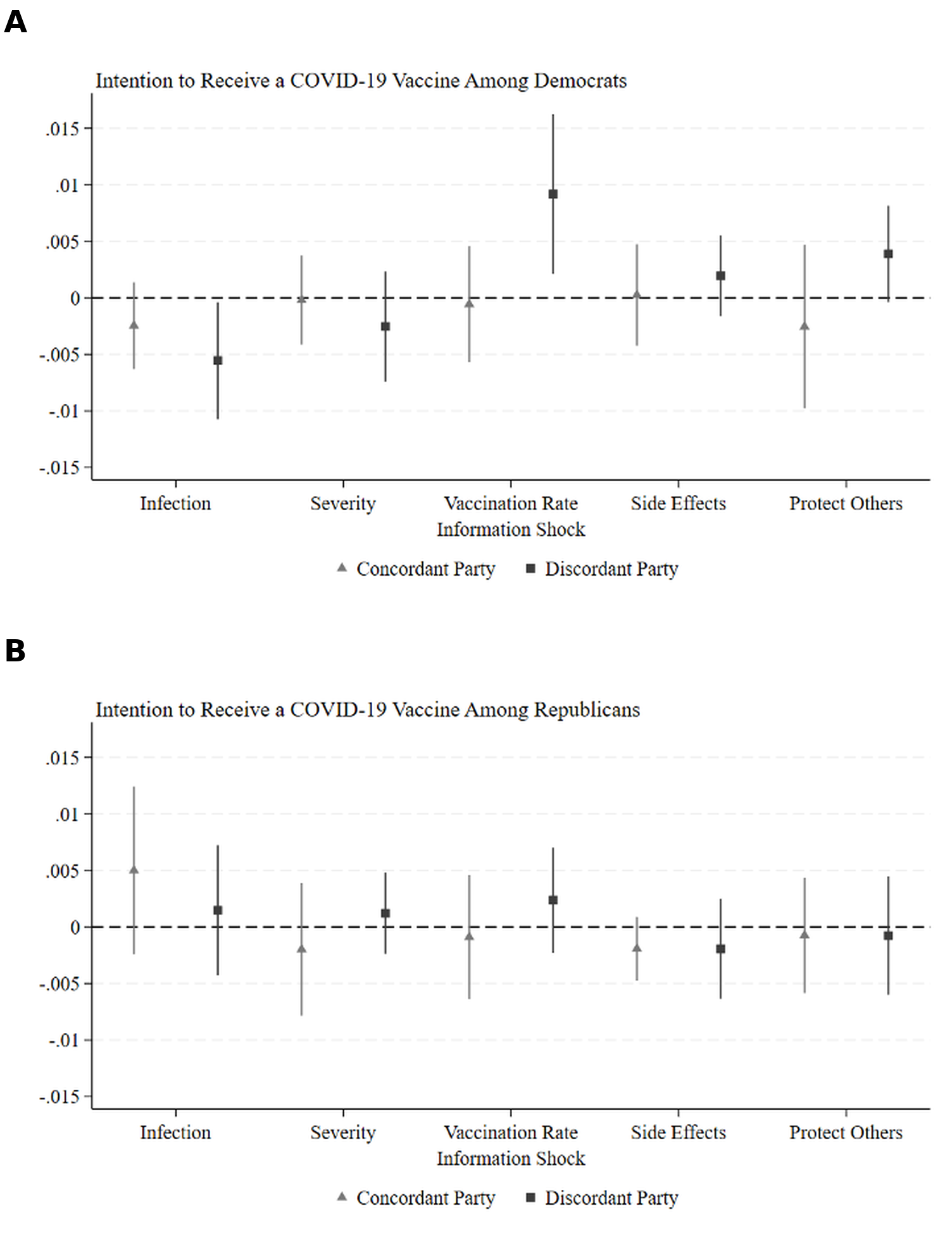

Supplement: S7 Fig — Panels A and B plots estimates obtained from a single regression whereby we interact our independent variables of interest with an indicator for each political party. The relationship is estimated via ordinary least squares. Panel A plots the estimates for Democrats while Panel B plots the estimates for Republicans. The darker grey triangles indicate the estimates from when an individual received a shock to their beliefs based on the experiences of someone with the same political party affiliation, while the lighter grey squares indicate the estimates from when an individual received a shock to their beliefs based on the experiences of someone with the opposite political party affiliation. The vertical lines denote the 95 percent confidence intervals. (TIF) [file pone.0352319.s008.tif]

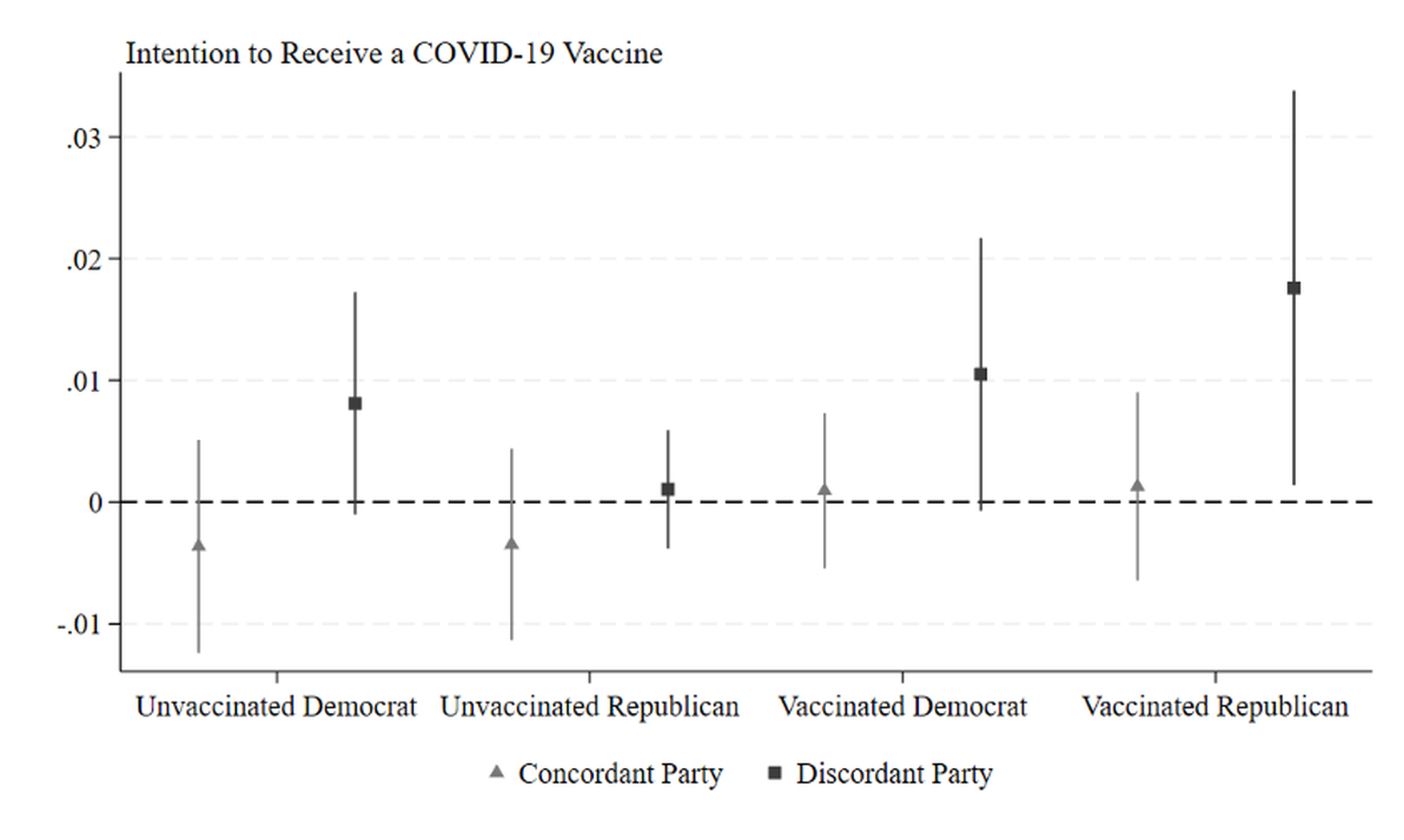

Supplement: S8 Fig — The figure plots estimates obtained from a specification our independent variables of interest in equation (2) with indicators for whether the participant was a Democrat or Republican and whether the participant reported previously receiving a COVID-19 vaccine. For brevity, we only report the estimates on the information shock of interest which measures the difference between participants’ initial beliefs and the revealed vaccination rate among members of the concordant or discordant political party. The estimates are obtained via ordinary least squares. The lighter grey triangles indicate the estimates from when an individual received a shock to their beliefs based on the experiences of someone with the same political party affiliation, while the darker grey squares indicate the estimates from when an individual received a shock to their beliefs based on the experiences of someone with the opposite political party affiliation. The vertical lines denote the 95 percent confidence intervals. (TIF) [file pone.0352319.s009.tif]
