# Supplementary material for: Partisan differences in healthcare decision-making: Evidence from a vaccine experiment
Source: PLoS One. 2026 Jul 20;21(7):e0352319. doi: 10.1371/journal.pone.0352319 (PMC13384293; doi:10.1371/journal.pone.0352319)
Supplement: S1 Table — Each panel reports the average difference between what the participants in our experiment predicted about the experiences of individuals with COVID-19 and the amount obtained in the pre-experiment survey. Standard deviations are reported in parentheses. Positive values indicate that on average participants overestimated a particular share, while negative values indicate that the participants underestimated the share. Panel A reports the average error when individuals in the full sample made predictions about the experiences of Democrats, and Panel B reports the average error when individuals in the full sample made predictions about Republicans. Panels C and D report these same figures but only among the subset of individuals who later had the actual values from the pre-experiment survey revealed to them. (DOCX) [file pone.0352319.s010.docx]

**S1 Table. Average Errors in Participants’ Beliefs About the Experiences**

**of Democrats and Republicans with COVID-19 During the Past 12 Months**

|  | (1) | (2) | (3) | (4) | (5) |
| --- | --- | --- | --- | --- | --- |
|  | Contracted COVID-19 | Sought Medical Intervention Conditional on Having a COVID-19 Infection | Received a COVID-19 Vaccine | Experienced Side Effects Conditional on Receiving a COVID-19 Vaccine | Reported Protecting Others as a Primary Reason for Receiving the COVID-19 Vaccine |
|  |  |  |  |  |  |
| **Panel A: Average Error in Beliefs About the Experiences of Democrats, Full Sample** | | | | | |
| Democrats | 25.15 | 20.15 | -0.85 | -37.85 | -26.85 |
|  | (25.07) | (25.07) | (25.07) | (25.07) | (25.07) |
|  |  |  |  |  |  |
| Republicans | 23.67 | 18.67 | 2.33 | -39.33 | -28.33 |
|  | (27.05) | (27.05) | (27.05) | (27.05) | (27.05) |
|  |  |  |  |  |  |
|  | | | | | |
| **Panel B: Average Error in Beliefs About the Experiences of Republicans, Full Sample** | | | | | |
| Democrats | 17.92 | 14.92 | 10.92 | -47.08 | -25.08 |
|  | (21.51) | (21.51) | (21.51) | (21.51) | (21.51) |
|  |  |  |  |  |  |
| Republicans | 19.23 | 16.23 | 12.23 | -45.77 | -23.77 |
|  | (23.28) | (23.28) | (23.28) | (23.28) | (23.28) |
|  |  |  |  |  |  |
|  | | | | | |
| **Panel C: Average Error in Beliefs About the Experiences of Democrats, Treated Group** | | | | | |
| Democrats | 32.43 | 31.37 | 15.46 | -26.00 | -0.842 |
|  | (26.32) | (24.83) | (26.81) | (25.24) | (18.13) |
|  |  |  |  |  |  |
| Republicans | 16.00 | 35.78 | 3.88 | -32.00 | -3.80 |
|  | (22.07) | (24.70) | (30.24) | (28.98) | (23.84) |
|  |  |  |  |  |  |
|  | | | | | |
| **Panel D: Average Error in Beliefs About the Experiences of Republicans, Treated Group** | | | | | |
| Democrats | 17.86 | 17.93 | 0.17 | -42.41 | -23.44 |
|  | (22.87) | (24.50) | (21.55) | (24.05) | (22.30) |
|  |  |  |  |  |  |
| Republicans | 9.88 | 20.30 | 5.34 | -39.53 | -16.93 |
|  | (19.31) | (23.09) | (24.09) | (26.20) | (25.14) |
|  |  |  |  |  |  |

Note: Each panel reports the average difference between what the participants in our experiment predicted about the experiences of individuals with COVID-19 and the amount obtained in the pre-experiment survey. Standard deviations are reported in parentheses. Positive values indicate that on average participants overestimated a particular share, while negative values indicate that the participants underestimated the share. Panel A reports the average error when individuals in the full sample made predictions about the experiences of Democrats, and Panel B reports the average error when individuals in the full sample made predictions about Republicans. Panels C and D report these same figures but only among the subset of individuals who later had the actual values from the pre-experiment survey revealed to them.
