# Supplementary material for: Partisan differences in healthcare decision-making: Evidence from a vaccine experiment
Source: PLoS One. 2026 Jul 20;21(7):e0352319. doi: 10.1371/journal.pone.0352319 (PMC13384293; doi:10.1371/journal.pone.0352319)
Supplement: S2 Table — The table reports the estimates obtained from equation (1), which examines the relationship between shocks to beliefs about COVID-19 and whether individuals reported that they intend to receive a COVID-19 vaccination in the next 12 months. Column 1 is estimated via ordinary least squares, while column 2 is estimated via logistic regression and reports the log odds ratios. (DOCX) [file pone.0352319.s011.docx]

**S2 Table. Baseline Relationship When
Not Accounting for Political Concordance**

|  | (1) | (2) |
| --- | --- | --- |
|  | Ordinary Least Squares | Logistic Regression |
|  |  |  |
| Share Contracting COVID-19 | -0.001 | -0.008 |
|  | (0.001) | (0.009) |
|  |  |  |
| Share of Infected with a Severe Infection | -0.000 | -0.001 |
|  | (0.001) | (0.008) |
|  |  |  |
| Vaccination Rate | 0.002 | 0.013 |
|  | (0.001) | (0.009) |
|  |  |  |
| Share with Side Effects from Vaccination | -0.001 | -0.005 |
|  | (0.001) | (0.006) |
|  |  |  |
| Share Vaccinating to Protect Others | 0.001 | 0.004 |
|  | (0.001) | (0.008) |
|  |  |  |
| R^2^ | 0.344 |  |
| Observations | 579 | 579 |
|  |  |  |

Note: The table reports the estimates obtained from equation (1), which examines the relationship between shocks to beliefs about COVID-19 and whether individuals reported that they intend to receive a COVID-19 vaccination in the next 12 months. Column 1 is estimated via ordinary least squares, while column 2 is estimated via logistic regression and reports the log odds ratios.
